# Experimentally-Derived Fibroblast Gene Signatures Identify Molecular Pathways Associated with Distinct Subsets of Systemic Sclerosis Patients in Three Independent Cohorts

**DOI:** 10.1371/journal.pone.0114017

**Published:** 2015-01-21

**Authors:** Michael E. Johnson, J. Matthew Mahoney, Jaclyn Taroni, Jennifer L. Sargent, Eleni Marmarelis, Ming-Ru Wu, John Varga, Monique E. Hinchcliff, Michael L. Whitfield

**Affiliations:** 1 Department of Genetics, Geisel School of Medicine at Dartmouth, Hanover, NH, United States of America; 2 Northwestern University Feinberg School of Medicine, Department of Internal Medicine, Division of Rheumatology, Chicago, IL, United States of America; University of Patras Medical School, GREECE

## Abstract

Genome-wide expression profiling in systemic sclerosis (SSc) has identified four ‘intrinsic’ subsets of disease (fibroproliferative, inflammatory, limited, and normal-like), each of which shows deregulation of distinct signaling pathways; however, the full set of pathways contributing to this differential gene expression has not been fully elucidated. Here we examine experimentally derived gene expression signatures in dermal fibroblasts for thirteen different signaling pathways implicated in SSc pathogenesis. These data show distinct and overlapping sets of genes induced by each pathway, allowing for a better understanding of the molecular relationship between profibrotic and immune signaling networks. Pathway-specific gene signatures were analyzed across a compendium of microarray datasets consisting of skin biopsies from three independent cohorts representing 80 SSc patients, 4 morphea, and 26 controls. IFNα signaling showed a strong association with early disease, while TGFβ signaling spanned the fibroproliferative and inflammatory subsets, was associated with worse MRSS, and was higher in lesional than non-lesional skin. The fibroproliferative subset was most strongly associated with PDGF signaling, while the inflammatory subset demonstrated strong activation of innate immune pathways including TLR signaling upstream of NF-κB. The limited and normal-like subsets did not show associations with fibrotic and inflammatory mediators such as TGFβ and TNFα. The normal-like subset showed high expression of genes associated with lipid signaling, which was absent in the inflammatory and limited subsets. Together, these data suggest a model by which IFNα is involved in early disease pathology, and disease severity is associated with active TGFβ signaling.

## Introduction

Systemic sclerosis (SSc) is a progressive fibrotic disease of unknown etiology characterized by fibrosis of the skin and internal organs, vascular abnormalities, immune activation, and excessive extracellular matrix deposition. Heterogeneity of disease symptoms and outcomes remains a significant obstacle, though emerging data are beginning to provide insight. Clinical classifications of SSc are based primarily on the extent of skin and internal organ involvement, and SSc autoantibody profiles [1]. Multiple high-throughput gene expression analyses of patient skin biopsies have identified four SSc intrinsic subsets that span the two clinically identified subsets of limited (lSSc) and diffuse (dSSc) disease. Distinct molecular signaling pathways appear to underlie each subset, providing insights into the clinically observed heterogeneity between SSc patients that has confounded clinical trials. Analysis of serial biopsies over 6–12 months has shown the intrinsic subsets to be stable over this short time frame, but does not rule out the possibility of patients changing subsets over much longer time scales [2].

Previously, we have described associations between both a TGFβ-responsive gene signature and increased disease severity in the fibroproliferative subset of dSSc patients [3], and an IL-13/CCL2 gene signature and the inflammatory subset [4]. While these associations were suggestive, the studies were limited by the small number of samples available, and the absence of a validation cohort. In addition, these pathways accounted for only a fraction of the overall gene expression present within each of the intrinsic gene expression subset of SSc. Here, we have expanded our analyses to include ten additional inflammatory and fibrotic signaling pathways (three experimentally derived here for the first time; seven taken from the literature), and expanded on two others, to determine the genes induced, the unique and overlapping genes among the pathways, and how each contributes to the gene expression changes in SSc skin. Along with our prior analyses of TGFβ, these pathway gene signatures were compared against three independent SSc patient cohorts, which were merged into a single dataset, and stratified into intrinsic gene expression subsets. This allows us to assess the relative contribution of each signaling pathway to the gene expression changes seen in SSc skin.

The list of pathways analyzed here includes both pathway analyses previously performed within our own group, along with pathways strongly implicated by the primary literature, but without knowledge of how they stratify across a sample of the SSc patient population. Pathways suggested by the literature include platelet-derived growth factor (PDGF), sphingosine-1-phosphate (S1P), peroxisome proliferator-activated receptor gamma (PPARγ), tumor necrosis factor alpha (TNFα), interferon alpha (IFNα), nuclear factor kappa-B (NF-κB), and innate immune signaling. The *in vivo* gene response to imatinib mesylate was also included in these analyses due to the overlapping functions of this drug, and its use as an experimental treatment for SSc [5].

IFNα signaling was strongly associated with early disease, while TGFβ signaling spanned both the inflammatory and fibroproliferative subsets, and was associated with more severe skin involvement. We find the fibroproliferative intrinsic subset to be more strongly associated with the PDGF gene signature, while the inflammatory subset is associated with a wide range of NF-κB activating pathways.

## Materials and Methods

### Skin biopsy data

Microarray data for scleroderma lesional and nonlesional skin biopsies and healthy controls used in this analysis have been described previously [2,6,7]. These data are publically available in the NCBI GEO database under accession numbers GSE9285, GSE32413, and GSE45485, respectively. Additional skin biopsy microarrays not previously described elsewhere are also included in this dataset, and are available from the NCBI GEO database under accession number GSE59785. The analysis of human samples in this study was approved by the Committee for the Protection of Human Subjects at Dartmouth College (CPHS# 16631) and by the institutional review boards (IRB) of Northwestern University’s Feinberg School of Medicine (STU00004428). All subjects in the study provided written consent, which was approved by the IRB review panels of Dartmouth College and Northwestern University Feinberg School of Medicine.

Batch effects evident between the three datasets were adjusted using ComBat [8] run as a GenePattern module using parametric and non-parametric settings. The statistical significance of batch bias before and after adjustment was assessed using guided principal component analysis (gPCA) and the first two unguided principal components were inspected (S1 Fig.). Genes were then selected using an intrinsic gene identifier algorithm [9] using a false discovery rate (FDR) sufficient to produce reproducible clusters (generally between 2000 and 3000 probes), clustered using Cluster 3.0 [10], and visualized with Java TreeView [11]. The distribution of intrinsic subset assignments in the original published datasets were compared to those determined after ComBat adjustment using a Chi-squared test.

### Experimental treatment and RNA preparation

Primary adult NHDFs were obtained from Cambrex Bioscience Inc. (East Rutherford, NJ); SSc fibroblasts were isolated from explanted lesional biopsies cultured for three passages in DMEM supplemented with 10% fetal bovine serum (FBS) (v/v) and 100 IU/mL penicillin-streptomycin. To measure pathway treatment responses, 4 × 10^5^ fibroblasts were seeded in 100 mm dishes, and cultured in DMEM supplemented with 10% FBS for 48 h; cells were then brought to quiescence in DMEM plus 0.1% FBS for 24 h. Cellular agonists (PDGF, R&D Systems, Minneapolis, MN; rosiglitazone (RZN), Cayman Chemical Company, Ann Arbor, MI; S1P, Sigma-Aldrich, St. Louis, MO; IL-4 and IL-13, Peprotech, Rocky Hill, NJ) were added to low serum media, and cells incubated for 0, 2, 4, 8, 12, and 24 h; baseline, zero hour time points were performed in triplicate. Following treatment, cells were lysed in RLT buffer supplemented with 0.1% β-mercaptoethanol, and total RNA isolated using RNeasy mini kits (Qiagen, Valencia, CA), according to the manufacturer’s instructions. Pathway gene signatures were defined as all probes exhibiting a ≥ 2-fold mean change in expression relative to controls at 12 and 24 h across all replicates. Data were filtered to include only probes showing an average correlation > 0.8 relative to an idealized induction pattern (full induction at 2–24 h time points).

### Quantitative real-time PCR

Reverse transcription of total RNA (200 ng) was performed using SuperScript II reverse transcriptase (Invitrogen, San Diego, CA) to generate single-stranded complementary DNA; 1.0 mg cDNA was used for each qRT-PCR reaction. Taqman gene expression probes for CD36, THBD, and 18S were obtained from Life Technologies (Foster City, CA), and analyzed using the 7500 Fast Real-Time PCR system. Fold changes were calculated relative to 18S controls using the comparative C_t_ formula 2^−∆∆Ct^ [12].Fold changes were calculated relative to 18S controls using the comparative C_t_ formula 2^−∆∆Ct^ [12]. All experiments were performed in triplicate.

### Microarray procedures

Microarray hybridizations were performed as described previously [7]. Briefly, RNA quality was assessed using the Agilent 2100 Bioanalyzer, and quantified using a Thermo Scientific NanoDrop 2000 spectrophotometer. Total RNA (200 ng) was amplified and labeled using Agilent QuickAmp Labeling kits, as described previously [6]. Cy3-labeled sample and Cy5-labeled Universal Human Reference RNA (Stratagene, La Jolla, CA) we co-hybridized onto Agilent SurePrint Human Genome 4 × 44k (G4112F) and 8 × 60k (G4851A) microarrays. Data were uploaded to the UNC microarray database, normalized, and filtered for spot quality and signal intensity. Microarray data from this paper have been deposited in the NCBI GEO database under accession numbers GSE56038 and GSE59785.

### Data analysis

Data analyses were performed for each of the 13 agonists: PDGF, S1P, RZN, TGFβ, IL-13, IL-4, IFNα, TNFα, Polyinosinic:polycytidylic acid (poly(I-C)), ionomycin-phorbol 12-myristate 13-acetate (ionomycin-PMA), dexamethasone (DEX), lipopolysaccharide (LPS), and imatinib mesylate. PDGF, S1P, and RZN time courses were performed as part of this analysis. TGFβ time courses were originally described by Sargent, *et al*. and are available from the NCBI GEO database under accession number GSE12493. Two additional IL-13 and IL-4 time courses each were performed adding to the data published in Greenblatt, *et al*. [4] and are available under accession number GSE56308. *In vitro* fibroblast treatment arrays for agonists IFNα, TNFα, poly(I-C), ionomycin-PMA, DEX, and LPS were originally described by Rubins, *et al.* [13], and are available from the NCBI GEO database under accession number GSE24125. *In vivo* imatinib mesylate treatment response microarrays were performed by Chung, *et al*. [5] using skin biopsies collected before and after treatment; these data are available from the NCBI GEO database under accession number GSE11130. A summary of all treatment-associated microarray data used in this study is presented in Table 1.

**Table 1:** Overview of agonists used in this study.

**Agonist**	**Primary Target**	**Source**	**Experimental Overview**	**Accession**
**PDGF**	**PDGFR**	**This study**	***In vitro* human fibroblast time course**	**GSE56308**
**S1P**	**S1PR1–5**	**This study**	***In vitro* human fibroblast time course**	**GSE56308**
**RZN**	**PPARγ**	**This study**	***In vitro* human fibroblast time course**	**GSE56308**
**IL-13**	**IL-13Rα1/IL-4Rα**	**This study**	***In vitro* human fibroblast time course**	**GSE56308**
**IL-4**	**IL-4Rα**	**This study**	***In vitro* human fibroblast time course**	**GSE56308**
**TGFβ**	**TGFβ**	**Sargent, *et al*., 2009**	***In vitro* human fibroblast time course**	**GSE12493**
**TNFα**	**TNFR1/2**	**Rubins, *et al*., 2011**	**24 h *in vitro* human fibroblast treatment**	**GSE24125**
**IFNα**	**IFNAR**	**Rubins, *et al*., 2011**	**24 h *in vitro* human fibroblast treatment**	**GSE24125**
**Poly(I-C)**	**TLR3**	**Rubins, *et al*., 2011**	**24 h *in vitro* human fibroblast treatment**	**GSE24125**
**LPS**	**TLR4**	**Rubins, *et al*., 2011**	**24 h *in vitro* human fibroblast treatment**	**GSE24125**
**DEX**	**Glucocorticoid receptors**	**Rubins, *et al*., 2011**	**24 h *in vitro* human fibroblast treatment**	**GSE24125**
**Iono-PMA**	**T cells/Ca^2+^/PKC**	**Rubins, *et al*., 2011**	**24 h *in vitro* human fibroblast treatment**	**GSE24125**
**Imatinib**	**Ableson kinase/PDGFR**	**Chung, *et al*., 2009**	**Skin biopsies from *in vivo* clinical trial collected before and after treatment**	**GSE11130**

Pathway gene signatures for each of the treatments listed in Table 2 were defined as all genes up or downregulated ≥ 2-fold across all of their corresponding 12 and 24 h time points, relative to untreated controls. The imatinib signature was determined based upon a *p*-value cutoff, as defined by Chung, *et al*. [5]. A centroid was created for each of the TGFβ, PDGF, S1P, RZN, IL-13, and IL-4 time courses by averaging the 12 and 24 h time points, and the centroids aligned to the MPH dataset using Agilent probe IDs. Data for IFNα, TNFα, poly(I-C), LPS, ionomycin-PMA, DEX, and imatinib were aligned by Entrez ID due to differences in microarray annotation; genes represented by multiple probes were averaged across all probes for both the treatment and MPH datasets. Pearson’s correlation coefficients were calculated between each pathway gene signature centroid and the MPH dataset for each individual microarray; average correlations were then calculated for each gene signature for each of the four intrinsic subsets. *P* values quantifying the enrichment of pathway signatures within individual subsets were calculated based upon the average Pearson’s correlation *r* coefficient using the standard method for Pearson’s correlation *P* value calculations, with *n* defined as the number of genes within each pathway.

**Table 2:** Experimental pathway gene signatures and overlap with the MPH dataset.

**Treatment**	**Number of probes passing filter ^a^, ^b^**	**Number of genes found in MPH dataset (% overlap) ^d^**
**PDGF**	**1198**	**728 (60.8)**
**TGFβ**	**946**	**842 (89.0)**
**S1P**	**848**	**825 (97.3)**
**IL-13**	**850**	**759 (89.3)**
**IL-4**	**1549**	**1415 (91.3)**
**RZN**	**222**	**128 (57.7)**
**LPS**	**1472**	**1185 (80.5)**
**PolyIC**	**4599**	**3749 (81.5)**
**TNFα**	**1487**	**1184 (79.6)**
**IFNα**	**262**	**223 (85.1)**
**Iono-PMA**	**3694**	**3040 (82.3)**
**Dex**	**1495**	**1151 (77.0)**
**Imatinib**	**1050 ^c^**	**843 (80.3)**

^a^ Pathway gene signatures were defined as all genes up or downregulated ≥ 2-fold across all 12 and 24 h time points, relative to untreated controls.

^b^ IDs for PDGF, TGFβ, S1P, IL-13, IL-4, and RZN denote unique Agilent probe IDs. Entrez gene IDs were used for LPS, PolyIC, TNFα, IFNα, Iono-PMA, Dex, and imatinib; all genes represented by two or more probes were averaged in both the MPH dataset and individual gene signatures.

^c^ The gene expression signature used for imatinib was determined based upon a p value cutoff, as defined by Chung, *et al*. [5].

^d^ MPH overlap signifies the number of genes IDs from a given pathway also appearing in the MPH dataset; the low overlap percentages seen in both PDGF and PPARγ pathways is a result of platform differences, as both PDGF and PPARγ pathways were reanalyzed on Agilent 8 × 60k DNA microarrays, while the MPH dataset includes only probes present in both 44k and 60k arrays.

Pearson’s correlation coefficients were used to quantify the contribution of a specific pathway to the gene expression within a given patient. These correlation ‘scores’ were then compared against clinically relevant factors including age, sex, modified Rodnan skin score (MRSS), biopsy site, and disease duration to identify the predictive value of each pathways for these clinical outcomes. Clinical comparisons were limited to dSSc patients only, using a single array per patient per time point collected; in cases where both lesional and non-lesional biopsies were collected only the lesional biopsy was considered. Comparisons of biopsy site were limited to clinically dSSc patients that provided paired lesional and non-lesional biopsies at a given time point; *n* denotes the number of patients included in each analysis. Continuous variables were compared using Pearson’s correlation; categorical variables were analyzed by ANOVA. All statistical analyses were performed using IBM SPSS version 19. *P* values ≤ 0.05 were considered statistically significant.

## Results

### Integrative analysis of the intrinsic subsets

*In vitro*, experimentally derived pathway signatures putatively deregulated in SSc provide an interpretive framework for previously generated skin biopsy data. Three distinct skin biopsy datasets consisting of 75 [6], 89 [2], and 165 ([7] and unpublished data) microarrays were merged using ComBat [8] to create a single microarray dataset (referred to as the MPH (Milano-Pendergrass-Hinchcliff) dataset). Together, these combined data include 329 microarray hybridizations from 287 unique biopsies representing 111 patients: 70 dSSc, 10 lSSc, 26 healthy controls, 4 morphea, and 1 eosinophilic fasciitis; one patient’s diagnosis changed from lSSc to dSSc during the period of study. This combined dataset was used as a reference against which the relative contributions of different signaling pathways could be compared in a genome-wide meta-analysis.

### Functional gene expression groups

**Figure 1:**
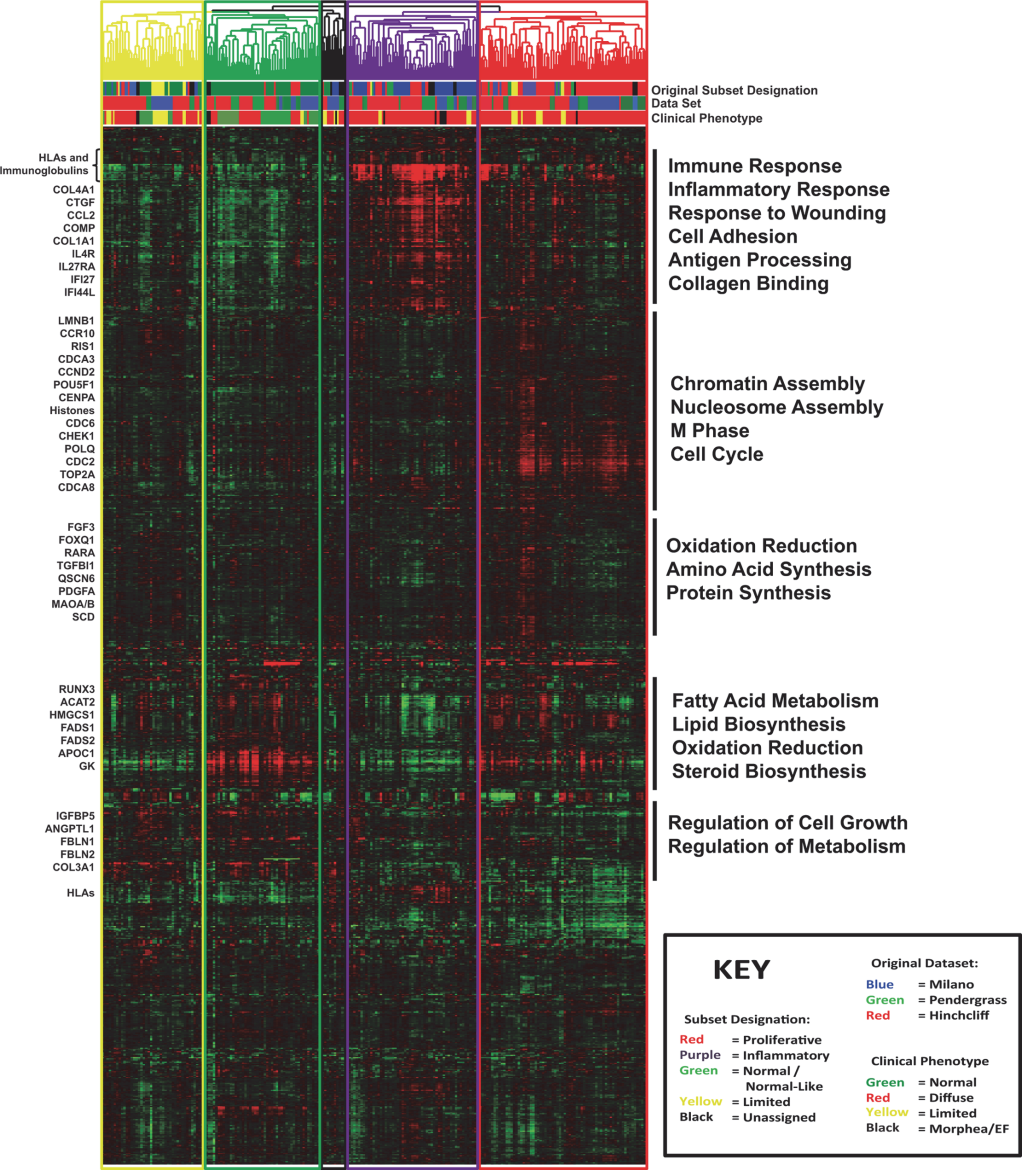
Hierarchical clustering recreates intrinsic subsets. Hierarchical clustering of the ComBat-merged MPH dataset recreates clear normal-like, fibroproliferative, inflammatory, and limited subsets. Clustering was performed on 2316 probes covering 2189 genes at an FDR of 0.65%, chosen based upon their consistent expression within an individual patient, along with high variance between patients. The array tree is color coded to indicate new intrinsic subset designations (yellow = limited, green = normal-like, purple = inflammatory, red = fibroproliferative, and black = unassigned). Below the array tree, hash marks are used to indicate the original subset designation (TOP: green = normal-like, red = fibroproliferative, purple = inflammatory, yellow = limited, black = unassigned), the dataset of origin (MIDDLE: blue = Milano, green = Pendergrass, red = Hinchcliff), and the clinical diagnosis (BOTTOM: green = normal, red = diffuse scleroderma, yellow = limited scleroderma, black = morphea or eosinophilic fasciitis). Black bars indicate genes that clustered together hierarchically, with the most highly represented GO terms listed alongside each cluster.

Clustering of the MPH dataset was performed as described previously [2,6,7], using the genes that showed the most intrinsic expression (e.g. the most consistent expression across all samples from a single patient but with the highest variance between different patients [9,14]). We selected 2316 probes covering 2189 unique genes at an estimated false discovery rate (FDR) of 0.65%. Average linkage hierarchical clustering was performed for both genes and arrays, recapitulating the four previously described ‘intrinsic’ subsets (fibroproliferative, inflammatory, limited, and normal-like; Fig. 1). A similar analysis performed using only a single array per patient revealed broadly similar results, indicating that the inclusion of multiple time points and technical replicates for some patients did not significantly affect the size of each subset (S2 Fig.).

As the MPH dataset is composed of previously described biopsy samples, the intrinsic subsets assignments identified in this analysis are similar to those previously described. The methods required to merge these three datasets into one group does cause some samples at the edges of groups to be misclassified [2,6,7]. Subset assignments were largely consistent between the original and MPH datasets (*p* ≤ 0.001, Chi-squared test; S1 Table). Strong reproducibility was observed in the inflammatory (47/75; 63%), and fibroproliferative (69/99; 70%) subsets where samples original classified as such were given the same classification here. The most common misclassification of fibroproliferative was to inflammatory (17 biopsies) and vice versa (16 inflammatory biopsies misclassified as fibroproliferative). Patients originally classified as the limited subset were typically classified as such here (12/19; 63%) and the most common misclassification was to fibroproliferative (6 biopsies). The normal-like subset showed the greatest variability (58/104 56%) with the majority of the misclassified samples (28 biopsies) being added to the limited dendrogram branch.

Gene clusters associated with each intrinsic subset were analyzed using the Database for Annotation, Visualization, and Integrated Discovery (DAVID) to identify functional enrichment. Gene ontology (GO) biological process annotations recapitulated those previously described. The inflammatory subset include *inflammatory respons*e, *immune response, cell adhesion, angiogenesis*, and *antigen processing* (*p* < 0.001; Fig. 1) and include multiple HLA and immunoglobulin genes, CTGF, CCL2, IL10RA, IL27RA, VEGFC, and genes associated with fibrosis (COMP, COL1A1, COL4A1, COL4A2, COL5A2, COL6A1, COL6A3, COL14A1, and COL15A1).

The fibroproliferative subset is significantly enriched for GO biological processes associated with the cell cycle including *chromatin assembly, nucleosome assembly, M phase*, and *cell cycle* (*p* < 0.001; Fig. 1), and includes genes for cell cycle regulators CCNE1, CDCA5, CDKN2A, and CCNB2, as well as multiple histone genes.

The normal-like and limited groups are defined primarily based upon the absence of immune or proliferation related gene expression, with the primary division between these groups driven by a strong a strong lipid and fatty acid metabolism signature in the normal-like group which is significantly decreased in the limited subset. This lipid signature is characterized by GO biological processes of *fatty acid metabolism, lipid biosynthesis, oxidation reduction*, and *steroid biosynthesis* (*p* < 0.001; Fig. 1). Genes principally involved in these processes include HMGCS1, fatty acid desaturases (FADS1 and FADS2), and acyl-CoA synthesis genes (ACADVL, ACAT2, ACOX2, and ACSL3).

### Generation of fibrotic pathway gene signatures in dermal fibroblasts

**Figure 2:**
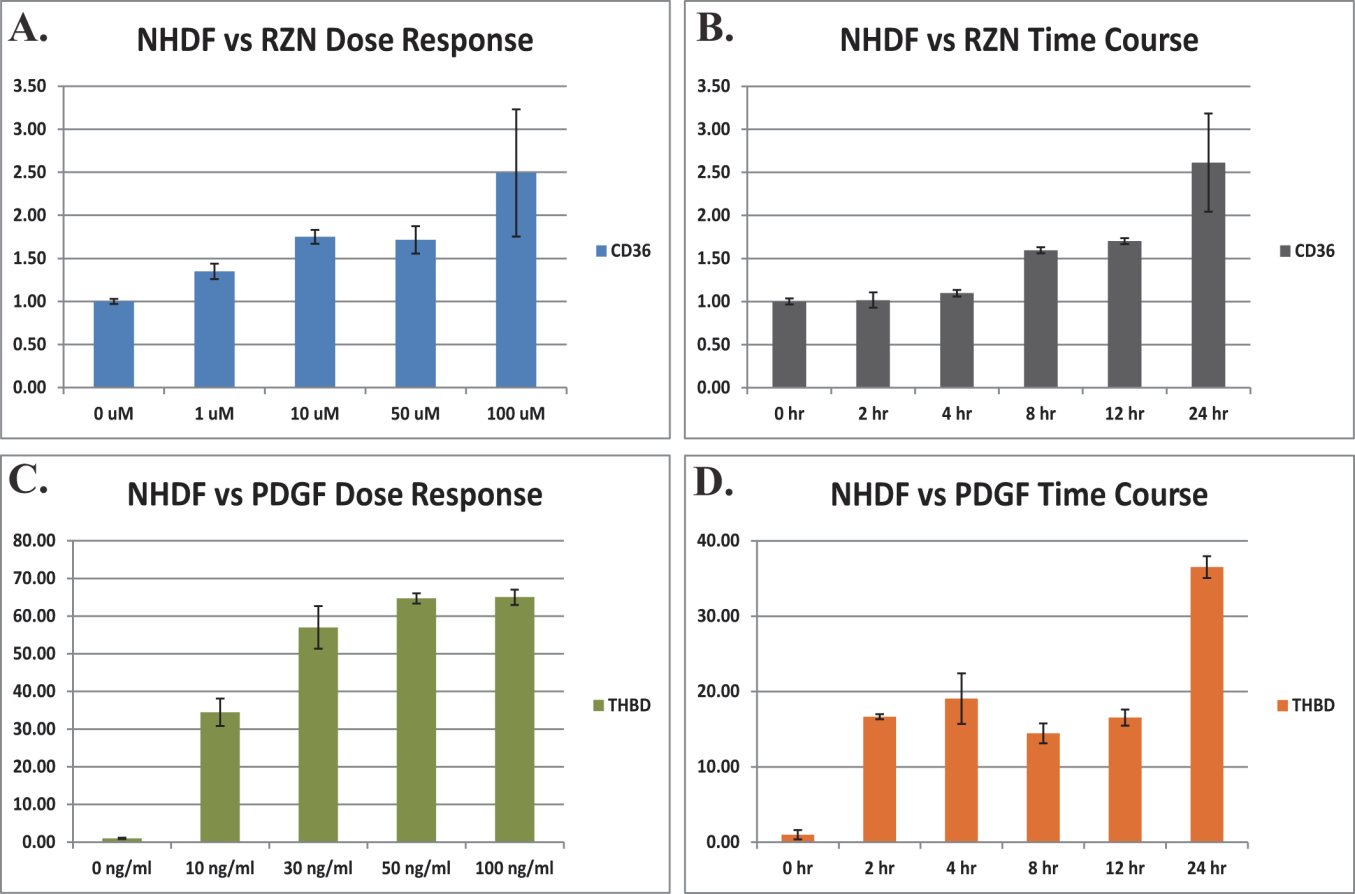
Dosage response and induction of reporter genes following stimulation with PDGF and RZN. RZN and PDGF concentrations were optimized for use in microarray treatment experiments by qRT-PCR using reporter genes CD36 and thrombospondin (THBD), respectively. NHDFs were treated with A) 0, 1, 10, 50, and 100 μM RZN or C) 0, 10, 30, 50, and 100 μg/mL PDGF for 24 h. Levels of CD36 and THBD were analyzed by qRT-PCR, and normalized to 18S rRNA. NHDFs were treated with B) 10 μM RZN and D) 30 ng/mL PDGF for 0, 0, 0, 2, 4, 8, 12, and 24 h. Error bars indicate the standard deviation across three or more replicates; all time points were statistically significant relative to controls (*p* < 0.05).

**Figure 3:**
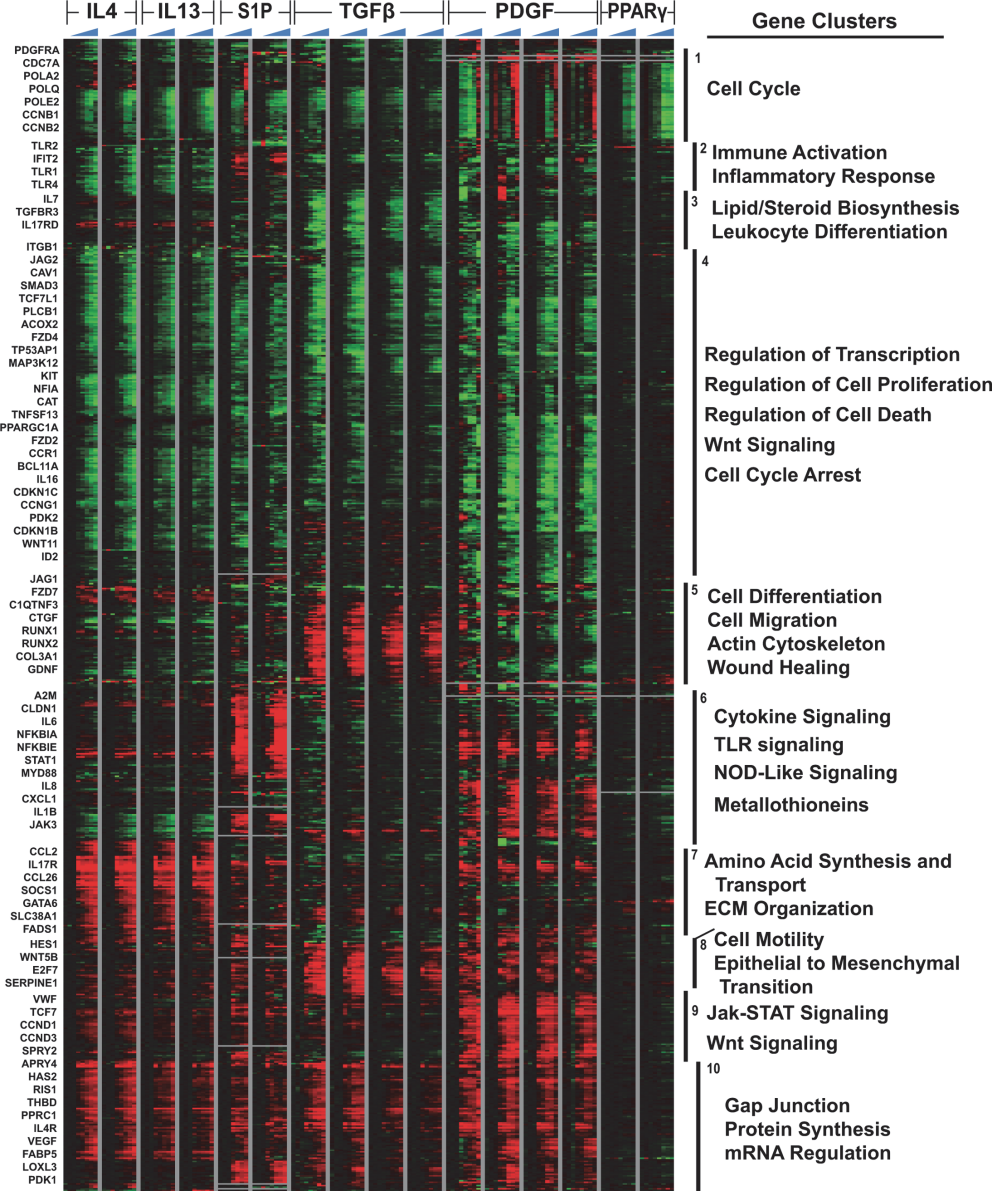
Clustering of pathway-regulated genes signatures reveals co-regulated and pathway specific modules. A total of 2136 probes covering 2081 genes were identified which show ≥ 2-fold average change in gene expression at 12–24 h in one or more of the six different pathways examined (IL-4, IL-13, S1P, TGFβ, PDGF, and RZN). Gene expression data from each of the eight time points (0, 0, 0, 2, 4, 8, 12, and 24 h) from each time course are shown. Black bars indicate genes that clustered together hierarchically, with the most highly represented GO terms listed alongside each cluster.

Using targets suggested by the literature, we performed treatment time courses for PDGF, S1P, and rosiglitazone (RZN), an agonist of PPARγ, in SSc and normal dermal fibroblasts to assess the role of each signaling pathway is SSc pathogenesis; we performed two additional time courses each for IL-4 and IL-13 to expand upon the work of Greenblatt *et al*. [4]. No significant differences were observed between the genes induced by the different treatments in SSc lesional and healthy control fibroblasts in culture, consistent with previous findings [3,15,16]. Optimal dosing for PDGF and RZN were determined experimentally (Fig. 2), with cellular responses measured by quantitative real-time PCR; dosing for S1P was chosen based upon published results [17,18]. A 10 μM concentration of RZN resulted in a 1.7-fold induction of CD36 (*p* < 0.001 vs. control), with only modest increases at higher concentrations (Fig. 2A). The gene expression response increased over the course of 24 h with 10 μM (Fig. 2B). Accordingly, we chose 10 μM for all RZN treatment time courses. Treatment with 30 ng/mL PDGF resulted in a 57-fold induction of thrombomodulin (THBD), with dosage above 50 ng/mL saturating (*p* < 0.001 vs. control; Fig. 2C). Based upon these results a concentration of 30 ng/mL was used for all PDGF time course experiments. THBD expression increased sharply upon treatment with PDGF, with maximal induction seen at 24 h (Fig. 2D). Table 1 provides a summary of the time courses generated in this study. Each time course was analyzed independently (see methods) and then all pathways concatenated into a single data file (Fig. 3).

We first examined the genes and biological processes affected by each pathway treatment independently. Since IL-4, IL-13, and TGFβ have been described previously [3,4], transcriptional responses to these agonists are not discussed in detail.

The link between PDGF signaling and SSc pathogenesis is well established [19–22], with many PDGF-mediated effects, including cell proliferation, abnormal vascular development, and immune activation [23] having important implications for SSc. PDGF treatment of dermal fibroblasts resulted in the induction or suppression of 1198 probes covering 1099 unique genes (Table 2; S3 Fig.). Genes with increased expression were highly enriched for GO biological processes related to kinase activity, phosphorylation, wound healing, cytokine signaling, and smooth muscle cell proliferation (*p* < 0.001). Specific genes include IL8R ligands (CXCL1, CXCL2, CXCL3), TNF receptor superfamily members (TNFRSF12A, TNFRSF21, TNFRSF6B, TNFRSF8), metallothioneins (MT1A, MT1B, MT1E, MT1L, MT2A), BCL2A1, CCL2, IFI44, and VEGF. Downregulated genes were enriched for GO biological processes associated with cell motility and migration, MAP kinase signaling, and Wnt receptor signaling. Genes downregulated by PDGF include CTGF, MAP3K8, and GATA6.

The lipid and fatty acid metabolism signature identified within the normal-like subset are indicative of increased PPARγ signaling, as suggested by Varga and coworkers [24–26]. PPARγ signaling exerts a potent anti-fibrotic response [27], and is antagonistic to TGFβ [25], suggesting a potential therapeutic role for this pathway in SSc. Activation of PPARγ signaling by RZN had only modest effects on fibroblasts in the absence of other signals. A total of 222 probes covering 219 unique genes were affected in this analysis, of which only 37 probes were upregulated including ADRP, ANGPTL4, and PDK4 (Table 2; S4 Fig.). Lowering of the 2-fold cutoff to 1.5-fold increased the overall number of probes to 985. This more permissive cutoff revealed enrichment for expected GO processes including regulation of lipid metabolism, lipid storage, and long-chain fatty acid synthesis (*p* < 0.05). GO biological processes for downregulated genes are almost exclusively associated with cell cycle regulation, including the terms M phase, cell cycle, mitosis, nuclear division, spindle organization, and others (*p* < 0.001); this result was seen with both 2 and 1.5-fold cutoffs.

S1P signaling has also been shown to play an important role in immune activation and regulation [28], with potent pro-fibrotic effects seen in both normal and SSc fibroblasts [29]. As S1P levels are regulated in part through TGFβ [18], this suggests both unique and overlapping functions associated with this pathway. S1P treatment induced the most diverse responses of any of the agonists tested, with ≥ 2-fold induction or suppression seen in 848 probes covering 749 unique genes (Table 2; S5 Fig.). Upregulated GO biological processes included immune activation, inflammatory and wounding responses, regulation of cell death, and proliferation (*p* < 0.001). Prominently induced pathways include IL8R, TGFβ, Toll-like receptor, PPAR, and VEGF signaling, along with substantial activation of interferon-inducible proteins, such as IFI44. Downregulated GO biological processes include metabolism of sugars (glucose, hexose, and monosaccharide), antigen processing and presentation, immune response, fatty acid synthesis, and cell adhesion (*p* < 0.001).

### Identification of specific and overlapping functions for each pathway

Significant overlap exists between pathway gene signatures, particularly for fibrotic genes, making it difficult to identify pathway-specific effects. To better delineate the genes induced by multiple pathways (common) and those induced by a single pathway (specific), all probes showing ≥ 2-fold change in expression across all 12 and 24 h time points were concatenated from each of our treatment pathways, and hierarchically clustered to identify functional gene clusters. Pathways included in this analysis were PDGF, RZN, and S1P, along with our expanded IL-4 and IL-13 time courses, and our previous data examining TGFβ-induced gene expression [3]. A total of 2136 probes covering 2081 genes were identified in one or more of the six pathways considered (Fig. 3); probes not present on both the 4×44k and 8×60k microarray platforms were excluded from this analysis.

The clustered data revealed several areas of divergence that may be important in the pathogenesis of SSc (Fig. 3). Cluster 1 is highly enriched for virtually all cell cycle associated genes present in this dataset and showed induction by PDGF at 12 and 24 h time points, while substantial downregulated was seen in all other pathways. Clusters 3 and 5 were most strongly associated with TGFβ signaling, exhibiting a strong decrease in lipid and steroid biosynthesis (Fig. 3, cluster 3), with increased expression of genes associated with cell differentiation, migration, and wound healing including CTGF and COL3A1; these genes were largely unaffected in the five other pathways tested.

Clusters 2 and 6 were selectively upregulated in S1P, exhibiting strong induction of multiple TLRs and interferon-inducible proteins, indicating a clear role for this pathway in innate immunity. Surprisingly, S1P showed a strong induction of the interferon-inducible proteins commonly observed in SSc and Lupus PBMC samples [30,31]. IL-8-related signaling (e.g. IL-8, CXCL1-3) was induced by both S1P and PDGF (Fig. 3, cluster 6), although PDGF lacked many of the other genes associated with innate immunity induced by S1P, including IL-6, NFKBIA, NFKBIE, TLR1, TLR2, and TLR4 (Fig. 3).

Cluster 7 was most strongly associated with IL-4/IL-13 signaling. GO terms associated with this cluster include Jak/STAT signaling, amino acid synthesis and transport, and extracellular matrix organization. CCL2 was among the genes highly upregulated in this cluster, consistent with previous findings [4]; however, increased CCL2 expression was also observed in S1P and PDGF treatments, illustrating that activation of multiple signaling pathways can induce CCL2 expression.

In addition to pathway-specific effects, substantial convergence of pathways was also observed. Gene expression patterns are highly similar in both IL-4 and IL-13 signaling pathways due to their convergence on the shared IL4RA receptor (Fig. 3). Pathway-specific variations exist, though modest to strong downregulation is seen throughout cluster 4 for IL-4, IL-13, S1P, TGFβ, and PDGF, while the same pathways show consistent upregulation in clusters 8 and 10. Cluster 8 is most strongly activated in TGFβ, and includes many of the biological responses associated with fibrogenesis, including robust induction of epithelial to mesenchymal transition, cell motility, and Wnt signaling; however, this cluster is also upregulated to varying degrees in IL-4, IL-13, S1P, and PDGF, suggesting widespread convergence on these genes typically associated with fibrosis. Cluster 10, is consistently upregulated by all six pathways and is characterized by induction of multiple cellular biological processes including protein complex synthesis and mRNA regulation.

Together these analyses identify important pathway-specific effects of each agonist, including wound healing, cell proliferation, and immune activation. Furthermore, these analyses provide important data regarding many of the genes associated with fibrosis, and shows their regulation by multiple pathways in dermal fibroblasts. A pdf containing the full data from Fig. 3 is available among the supplemental materials (S6 Fig.).

### Curation of NF-κB-related signaling pathways and the imatinib response signature

Next, additional microarray data probing the response of dermal fibroblasts to a wide range of immunological perturbations were downloaded from the NCBI GEO database (Table 1). These pathways are particularly relevant to SSc due to the inflammatory gene expression observed in our skin biopsy dataset. *In vitro* fibroblast treatment data were obtained for TNFα, IFNα, lipopolysaccharide (LPS), polyinosinic-polycytidylic acid (poly(I-C)), ionomycin plus phorbol-12-myristate-13-acetate (ionomycin-PMA), and dexamethasone (DEX), [13]. TNFα and IFNα are among the first cytokines expressed during an innate immune response, and are important for the generation of adaptive T cell responses [32]. TNFα plays a major role in both acute and chronic inflammation [33], while IFNα acts as an important mediator of antiviral activity [34]. Both LPS and poly(I-C) initiate innate immune responses through Toll-like receptors, activating TLR4 and TLR3, respectively. Ionomycin-PMA raises intracellular Ca^+^ levels, and induces protein kinase C (PKC) activation [35,36]. DEX is a synthetic glucocorticoid steroid which functions as a potent anti-inflammatory. Due to differences in platforms, gene annotation, and experimental design, microarray data from each of these treatments were processed independently; genes represented by multiple probes were averaged across all probes for both the treatment and MPH datasets. Each set of genes constitutes a ‘signature’ for that pathway.

The final set of data included in this study was taken from a case report study performed by Chung, *et al*. [5] examining the effect of imatinib mesylate on two dSSc patients. Imatinib is a selective tyrosine kinase inhibitor which blocks phosphorylation of Abelson kinase (Abl), a target of both TGFβ and PDGF, as well as the PDGF receptor (PDGFR). Microarray analyses were performed using skin biopsies collected before and after treatment, with the imatinib response signature determined based upon a *p*-value cutoff. We used the list of 1050 imatinib response genes as published by Chung *et al*. [5] (Table 1).

### Contributions of individual pathways within each intrinsic subset of disease

**Figure 4:**
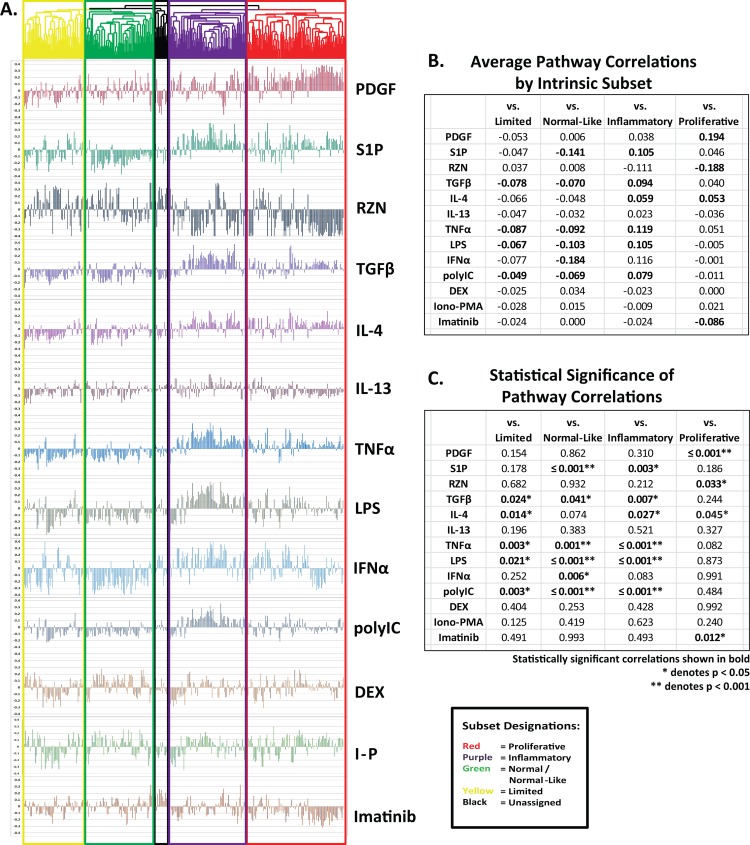
Correlations between pathway-specific gene signatures and patient gene expression profiles. Pearson correlations were performed between each of the thirteen pathway-specific gene signatures and the corresponding probes in the MPH dataset. A. Pathway gene signatures are defined as all probes exhibiting ≥ 2-fold average change in gene expression across all 12 and 24 h time points for a given treatment (Table 2). Correlations were repeated across each of the 329 arrays and aligned using the array dendogram from Fig. 1. Boxes representing each of the four intrinsic subsets (normal-like = green, fibroproliferative = red, inflammatory = purple, limited = yellow) are shown; arrays not clustering with any defined subset are indicated in black. B. Average Pearson’s correlations for each pathway across each of the intrinsic subsets are provided. C. *P* values quantifying the enrichment of pathway signatures within individual subsets were calculated based upon the average Pearson’s correlation, with statistically significant correlations highlighted in bold.

To identify the contribution of each pathway to the overall gene expression profile observed in patient biopsies, Pearson’s correlations were performed comparing each of the thirteen gene expression signatures against the corresponding probes extracted from the MPH skin biopsy dataset. Due to differences in DNA microarray platforms, not every probe or Entrez gene ID induced by a pathway was present in the MPH dataset. The number of probes and Entrez gene IDs for each pathway, and the corresponding number present in the MPH dataset are shown in Table 2. Correlations were then averaged for each intrinsic gene expression subset, as indicated by the colored coded dendrogram (Fig. 4A).

The relative contributions of each of the thirteen pathways to the differential gene expression that defined the four intrinsic subsets are represented by their average Pearson’s correlation scores (Fig. 4B) and associated *p* values (Fig. 4C). The strongest correlation between any intrinsic subset and pathway signature was seen between the fibroproliferative subset and PDGF (average correlation = 0.194; *p* < 0.001; Fig. 4B and C); the only other pathway showing a significant positive correlation to the fibroproliferative subset was IL-4, which exhibited modest, but consistent activation across the inflammatory subset, and variable activation in the fibroproliferative subset (average correlation = 0.053; *p* = 0.045; Fig. 4B and C). Surprisingly, the average TGFβ correlation for the entire fibroproliferative subset was only 0.040 (*p* = 0.244; Fig. 4B and C), indicating that sustained TGFβ expression is variable across patients in this subset. This observation was surprising however, given previous results obtained using the same TGFβ time courses and skin biopsy microarray data solely from Milano, *et al*. [3]. The fibroproliferative subset of arrays originally described in Milano, *et al*. does continue to show a positive correlation with TGFβ, however this average correlation is significantly lower than that seen with PDGF (TGFβ average correlation = 0.088; *p* = 0.011 vs. PDGF average correlation = 0.258; *p* < 0.001). RZN exhibited a significant negative correlation to this subset (average correlation = −0.188; *p* = 0.033; Fig. 4B and C), due primarily to the strong downregulation of cell cycle genes seen in this pathway.

The data of Chung *et al*. [5] suggested that dSSc patients in the fibroproliferative subset were most likely to benefit from tyrosine kinase inhibitor therapy. A direct comparison of the imatinib response signature to the MPH dataset supports this view, with a significant negative correlation evident for the fibroproliferative subset (average correlation = −0.086; *p* = 0.012; Fig. 4B and C). Imatinib also shows a strong negative correlation to the PDGF gene signature (= −0.824); this correlation is significantly stronger than with TGFβ (= −0.220), suggesting that the PDGF gene expression signature may also be a good predictor of response to imatinib.

The inflammatory subset is positively associated with the widest array of pathway signatures, likely due to the convergence of many of these pathways at NF-κB. The LPS, TNFα, S1P, poly(I-C), and TGFβ gene signatures are all significantly enriched within this subset (*p* < 0.001 for all; Fig. 4B and C). The IFNα gene signature is also enriched in this subset, though this correlation fails to reach statistical significance due to the relatively small number of genes in this pathway (average correlation = 0.116; *p* = 0.083). Enrichment of TGFβ signaling within this subset was surprising based on our prior findings; from these data it suggests that TGFβ signaling spans both the inflammatory and fibroproliferative subsets. TGFβ has been shown to induce both pro- and anti-inflammatory responses depending upon the presence of other cytokines [37], and can activate NF-κB by means of TGFβ-associated kinase 1 (TAK1) [38,39]. The strong correlation between S1P and the inflammatory subset, but not the fibroproliferative subset, was also surprising given the well documented roles for S1P in fibrosis, cell proliferation, and immune activation. Evidence from these analyses indicates a much stronger role for S1P in immune activation in SSc. Combined, these correlations suggest a role for innate immune signaling through NF-κB as an important mediator of pathology within the inflammatory subset.

Consistent with our prior studies, both the IL-4 and IL-13 gene signatures are associated with the inflammatory subset. The IL-4 pathway is significantly enriched suggesting a role for T_H_2-like immune responses (average correlation = 0.059; *p* = 0.027; Fig. 4B and C) in this subset. Despite its strong correlation with IL-4 (= 0.942; *p* < 0.001), the IL-13 signature initially showed only weak correlation to this subset (*p* = 0.521); however this difference was largely an artifact of 2-fold cutoff, as the IL-4 signature is almost twice the size of the IL-13 signature (1415 genes for IL-4 vs. 759 genes for IL-13). An equivalently sized 1415 gene signature from the IL-13 treatment showed enrichment in the inflammatory subset, although this correlation failed to reach statistical significance (*p* = 0.101).

The limited and normal-like subsets show very similar gene expression, exhibiting negative correlations to almost all of the pathways tested. These negative correlations were particularly strong among the pathways activated in the inflammatory subset; S1P, TGFβ, TNFα, LPS, and poly(I-C) (*p* < 0.05 for all; Fig. 4B and C), indicative of a more immunologically quiescent form of disease. The primary distinction between the two subsets was the high level of gene expression associated with lipid signaling in the normal-like subset. Surprisingly, the RZN gene signature exhibited no enrichment within this subset despite being an agonist for many of the upregulated genes. This absence of correlation is likely due to the low number of genes positively affected by RZN in the fibroblast, indicating that that fibroblasts are not the primary source of lipid signaling seen in these patients.

### TGFβ is associated with increased disease severity while IFNα is associated with early disease

Pearson’s correlations for each of the thirteen pathways were compared against clinically relevant factors including age, sex, skin score (MRSS), biopsy site, and disease duration to identify specific associations between individual pathways and disease outcomes. Clinical variables including lung disease, gastrointestinal involvement, renal disease, Raynaud’s severity, race, and autoantibody profile were not considered due to insufficient data across the multiple skin biopsy cohorts analyzed. Clinically limited SSc, morphea, and eosinophilic fasciitis patients were excluded from this analysis due to underlying differences in MRSS, age, and disease duration between clinical subsets, which limited to the analysis solely to dSSc patients. We limited the analysis to a single microarray per patient per time point collected; in cases where both lesional and non-lesional biopsies were collected only the lesional biopsy was considered.

Multiple signaling pathways exhibited strong correlations with MRSS (Table 3). Of the six agonists with significant correlation to MRSS (S1P, RZN, IL-4, TGFβ, TNFα, and imatinib), TGFβ was by far the strongest overall predictor of severity of skin involvement, with a correlation score nearly double that of the next highest pathway (average Pearson’s correlation = 0.385 for TGFβ vs. 0.210 for IL-4).

**Table 3:** Associations with clinical outcomes.

		**IL-4**	**IL-13**	**PDGF**	**RZN**	**S1P**	**TGFβ**	**DEX**	**IFNα**	**Iono-PMA**	**LPS**	**PolyIC**	**TNFα**	**Imatinib**
**mRSS**	**Pearson**	0.210	−0.108	0.134	−0.210	0.183	0.385	0.095	−0.057	0.073	0.117	0.098	0.179	−0.182
***n* = 116**	***P* value**	0.023*	0.136	0.064	0.004*	0.011*	≤ 0.001*	0.189	0.431	0.312	0.106	0.178	0.013*	0.011*
**Age**	**Pearson**	0.003	0.040	−0.106	0.069	−0.017	−0.038	−0.054	0.034	−0.033	0.033	0.025	−0.023	0.203
***n* = 116**	***P* value**	0.979	0.584	0.144	0.344	0.812	0.601	0.461	0.641	0.647	0.654	0.730	0.749	0.005*
**Disease Duration**	**ANOVA**	2.386	0.372	0.868	0.453	2.141	0.084	2.998	5.098	2.240	0.075	0.145	1.313	3.067
***n* = 115**	***P* value**	0.125	0.543	0.354	0.502	0.146	0.773	0.086	0.026*	0.137	0.785	0.704	0.254	0.083
**Sex**	**ANOVA**	0.002	1.451	0.150	0.013	0.278	0.480	0.201	0.428	0.749	0.590	0.768	0.142	0.060
***n* = 116**	***P* value**	0.963	0.230	0.699	0.910	0.599	0.489	0.654	0.514	0.388	0.443	0.382	0.707	0.806
**Biopsy Site**	**ANOVA**	0.012	0.335	3.962	2.325	0.613	12.170	0.986	3.311	2.273	0.719	1.476	0.551	7.097
***n* = 152**	***P* value**	0.914	0.564	0.048*	0.129	0.435	0.001*	0.322	0.071	0.134	0.398	0.226	0.459	0.009*

Pearson’s correlations comparing each of the arrays and pathways tested were used to quantify the overall contribution of a given pathway within an individual patient. These scores were then compared against clinically relevant factors including age, sex, modified Rodnan skin score (MRSS), biopsy site, and disease duration to assess the predictive value of each pathway for disease outcomes. Early disease was defined as disease ≤ 2 years after first non-Raynaud’s phenomenon symptoms. Comparisons were performed with clinically diffuse patients only, using a single array per patient for each time point collected. Comparisons of biopsy site were limited to clinically diffuse patients which provided paired lesional and non-lesional biopsies at a given time point; *n* denotes the number of patients included in each analysis. Continuous variables were compared using Pearson’s correlation; categorical variables were analyzed by ANOVA.

* denotes p < 0.05.

In addition to MRSS, the TGFβ gene signature was also strongly associated with biopsy site, showing a significant increase in TGFβ activation in lesional skin (average correlation = 0.058 vs. 0.002 in forearm and back, respectively; *p* = 0.001; Table 3). Alternatively, PDGF signaling appears elevated in non-lesional back skin (average correlation = 0.126 vs. 0.078 in back and forearm, respectively; *p* = 0.048; Table 3). These observations suggest subtle, but reproducible differences between lesional and unaffected skin [2,6,15], and may reflect differences between TGFβ and PDGF-driven disease.

Disease duration showed a significant negative correlation to IFNα pathway activation (Table 3), indicating a spike in IFNα signaling early in disease pathogenesis (defined as ≤ 2 years after first non-Raynaud’s symptoms), followed by downregulation of this pathway as disease progresses. Other inflammatory signals, including S1P and IL-4 were also higher in early disease though these signals did not reach statistical significance (p > 0.05; Table 3).

Finally, comparisons between the inflammatory and fibroproliferative subsets are suggestive of a weak association between disease stage and subset, with the fibroproliferative subset containing 8 of 11 patients with disease lasting ≥ 8 years, though this enrichment was not statistically significant (*p* = 0.104 by Chi-squared test). Both age and sex were comparable between subsets.

Taken together, these data suggest that IFNα signaling and other immune activation pathways may play a role in early disease pathogenesis, while TGFβ signaling is most strongly associated with disease severity. The observation that TGFβ spans the inflammatory and fibroproliferative subsets suggests a mechanistic connection may exists between these groups, driven in part by TGFβ signaling.

## Discussion

Scleroderma is a clinically heterogeneous disease that is likely to be caused by a network of pathways with distinct and overlapping effects. One way of determining the degree to which each pathway contributes to disease pathogenesis is to have a list of genes induced by each pathway in the primary cell type of interest, dermal fibroblasts. The data presented here provide a framework by which we can query and dissect the molecular signaling pathways that contribute to each of the intrinsic subsets.

The inflammatory subset shows strong positive correlations with a wide array of signaling pathways, with significant overlap in the induced genes. The most obvious point of convergence is NF-κB, a signaling intermediary shared between LPS, poly(I-C), IFNα, TNFα, S1P, and TGFβ. Indeed, many of these pathways appear to be directly linked in SSc; TLR signaling was found to induce strong upregulation of both type I IFNs (IFNα and IFNβ) and TGFβ in SSc skin and fibroblasts [40], providing a mechanism through which these signals may be linked. This convergence on TLRs and NF-κB is consistent with reports implicating innate immune activation in SSc pathogenesis [41].

In addition to NF-κB-mediated signaling, activation of other pathways within the inflammatory subset suggests distinct cell populations that may contribute to SSc pathology, providing hypotheses that can be tested experimentally. Strong IL-4-related gene expression in the inflammatory subset is consistent with T_H_2-like immune responses in these patients. Combined with the clear co-occurrence of TGFβ and innate immune signals, these data suggest a central role for alternatively activated (M2) macrophages in the inflammatory subset of SSc. M2 macrophages are known to be induced by a combination of T_H_2 cytokines, such as IL-4 and IL-13, in combination with TGFβ [42], and likely play key roles in SSc pathogenesis. Evidence for M2 macrophages has been observed in SSc lesional skin [43], lung [44–46], and serum [47], showing that these cells are likely to be involved in the initiation of fibrosis.

In addition to T_H_2-like immune responses, growing evidence suggests a role for T_H_17 cells in the pathogenesis of SSc with clear differences between diffuse and limited disease [48–52]. T_H_17-like immune responses have been implicated in a wide range of autoimmune conditions, including multiple sclerosis, systemic lupus erythematosus, psoriasis, neuromyelitis optica, Crohn’s disease, inflammatory bowel disease, and rheumatoid arthritis, suggesting a common mechanism of pathology associated with autoimmunity [53–56]. Parallels drawn between SSc and other autoimmune diseases may help to explain some of the contradictory signals seen in SSc, including activation of type I IFNs within the inflammatory subset. Under normal conditions type I IFNs are potent inhibitors of T_H_17 activity [51]; however, in many autoimmune diseases these signals actually enhance T_H_17 responses, exacerbating disease [53]. While the TGFβ and TNFα gene expression signatures seen in some patients in the inflammatory subset, in conjunction with pervasive inflammatory infiltrates, are consistent with a T_H_17-like immune response [37], additional pathway analyses examining other cytokines, such as IL-6 and IL-17, will be necessary to determine the relative contribution of T_H_17-like responses in each of the intrinsic subsets, as well as the presence of these signals over time.

Analysis of clinical covariates revealed a clear association between the TGFβ gene signature and increased MRSS severity, consistent with previous findings [3]. The strong association between the TGFβ gene signature and clinically affected forearm skin likely reflects the increased fibrosis at these sites.

The gene expression signature most strongly associated with the fibroproliferative subset was PDGF, which was not measured in our prior work [3]. The association is driven primarily by the strong upregulation of cell cycle and other proliferation-related genes, in contrast to TGFβ, which is traditionally regarded as an inhibitor of cell proliferation. Emerging evidence suggests that TGFβ signaling may span the inflammatory and fibroproliferative subsets, providing a potential mechanistic link between these two groups (Mahoney *et al.*, Submitted). If we were to consider an interpretation of the intrinsic subsets as mechanistic stops in disease progression rather than independent groups, expression of TGFβ during the initial inflammatory phase may play a role in the initiation of fibrosis, while sustained expression of TGFβ may induce greater expression of PDGF [57], leading to a more proliferative form of disease.

In addition to TGFβ, the timing of IFNα signaling may play a role in regulating the transition from the inflammatory to fibroproliferative subset. Under certain conditions, type I interferons are capable of inhibiting both PDGF activation and PDGF-mediated collagen expression [58]. Downregulation of IFN signaling would remove these inhibitory signals, hastening the transition to a more PDGF-driven, proliferative form of disease. Such a process may explain some of the negative treatment outcomes associated with anti-IFNα therapy in SSc, including a worsening of disease symptoms following therapy [59]. Such an outcome highlights the need for a better understanding of the interrelationship of SSc associated pathways, how they may change during disease progression, and if combination therapies could more effectively stop disease progression.

Beyond the actions of TGFβ alone, the maintenance and progression of fibrotic phenotypes has been shown to be driven in part by the mechanical environment [60]. Specific evidence regarding this phenomenon has recently been extended to SSc, with changes in the cell-matrix sufficient to perpetuate pro-fibrotic responses, even in the absence of other stimuli [61]. As heightened matrix stiffness has been shown to increase signaling through PDGFR [62], this suggests a mechanism by which physical changes in affected tissues can perpetuate disease after the initial inflammation has been resolved. Clearance of inflammation alone may therefore be insufficient for resolving disease phenotypes.

Patients clustering to the limited and normal-like subsets exhibited near-zero to negative correlations against all thirteen agonists tested, indicative of a non-proliferative, immunologically quiescent state of disease. Further longitudinal studies will be necessary to determine how these patients progress from a clinical standpoint, and whether they transition into another more active subset of disease over time.

One possible model suggested by our analysis of patient biopsy data is that of a cascade of signaling pathways generating the progressive disease we know as SSc. A progressive model of pathogenesis, in which each intrinsic subset represents a distinct phase of disease progression, provides the simplest interpretation of the data. A weakness of this model is that we have not been able to capture patients changing subsets when analyzing patients longitudinally over 6 to 12 months. However, this could simply mean that patients move between intrinsic subsets very slowly over time or in a way that is difficult to capture experimentally with longitudinal biopsies.

Direct validation of this progressive model of disease pathogenesis has not been performed due to the absence of appropriate model systems, and the duration of time necessary to observe these changes in patients; however, all of the agonists and cell types implicated in this model have been well documented in SSc. Agonists such as TGFβ [3,63,64], PDGF [20,65], IL-4 [66], IL-13 [4,66], IFNα [67], S1P [68], and TNFα [69] are present in the skin, sera, and bronchoalveolar fluid of SSc patients, while cell types such as M2 macrophages [43,45] and T_H_2 cells [70] have also been described. While considerable effort will be necessary to validate such a model, it provides a framework from which to link seemingly divergent observations into a single, comprehensive model of disease pathogenesis. Longitudinal studies examining gene expression and cytokine profiles, along with direct confirmation of the cell types involved in each step, will be necessary to clearly define the processes underlying each stage of disease progression.

## Supporting Information

S1 Fig Principal Component Analysis of Merged Datasets. The statistical significance of batch bias before and after adjustment was assessed using guided principal component analysis (gPCA) and the first two unguided principal components were inspected. The proportion of the variance associated with each unguided principal component is labeled on the axes. *P* values ≤ 0.05 are indicative of significant batch bias.
(EPS)

S2 Fig Hierarchical clustering recreates intrinsic subsets. Hierarchical clustering of the ComBat-merged MPH dataset recreates clear normal-like, fibroproliferative, inflammatory, and limited subsets. Clustering was performed on 2316 probes covering 2189 genes at an FDR of 0.65%, chosen based upon their consistent expression within an individual patient, along with high variance between patients. The array tree is color coded to indicate new intrinsic subset designations (yellow = limited, green = normal-like, purple = inflammatory, red = fibroproliferative, and black = unassigned). Below the array tree, hash marks are used to indicate the original subset designation (TOP: green = normal-like, red = fibroproliferative, purple = inflammatory, yellow = limited, black = unassigned), the dataset of origin (MIDDLE: blue = Milano, green = Pendergrass, red = Hinchcliff), and the clinical diagnosis (BOTTOM: green = normal, red = diffuse scleroderma, yellow = limited scleroderma, black = morphea or eosinophilic fasciitis). Black bars indicate genes that clustered together hierarchically, with the most highly represented GO terms listed alongside each cluster.
(EPS)

S3 Fig Hierarchical clustering of PDGF time courses. Normal human dermal fibroblasts and SSc-derived dermal fibroblasts were treated with 30 ng/mL PDGF, with samples harvested at 0, 2, 4, 8, 12, and 24 h. Data shown include all probes exhibiting ≥ 2-fold change in expression relative to untreated controls across all 12 and 24 h time points. Genes were clustered using Cluster 3.0, and visualized with Java TreeView.
(EPS)

S4 Fig Hierarchical clustering of RZN time courses. Normal human dermal fibroblasts were treated with 10 μM RZN, with samples harvested at 0, 2, 4, 8, 12, and 24 h. Data shown include all probes exhibiting ≥ 2-fold change in expression relative to untreated controls across all 12 and 24 h time points. Genes were clustered using Cluster 3.0, and visualized with Java TreeView.
(EPS)

S5 Fig Hierarchical clustering of S1P time courses. Normal human dermal fibroblasts and were treated with S1P, with samples harvested at 0, 2, 4, 8, 12, and 24 h. Data shown include all probes exhibiting ≥ 2-fold change in expression relative to untreated controls across all 12 and 24 h time points. Genes were clustered using Cluster 3.0, and visualized with Java TreeView.
(EPS)

S6 Fig Searchable version of Fig. 3. A searchable version of Fig. 3 including gene names for all probes exhibiting a ≥ 2-fold average change in gene expression at 12–24 h in one or more of the six different pathways examined.
(EPS)

S1 Table Patients included in this study. Full clinical data and associated pathway correlation scores for all patients and biopsies included in this study.
(XLS)

S2 Table 2-fold IDs for all pathways included in this study. Agilent probe IDs and Entrez gene IDs of all genes up- or downregulated ≥ 2-fold across all 12 and 24 h time points for each pathway tested.
(XLSX)
